# Seroprevalence of *Bordetella pertussis* infection in children 1–14 years old: Indonesia basic health research (Riskesdas) 2013 and 2018 data

**DOI:** 10.1371/journal.pone.0311362

**Published:** 2024-09-30

**Authors:** Monica Dwi Hartanti, Novaria Sari Dewi Panjaitan, Sunarno Sunarno, Nathalia Ningrum, Armedy Ronny Hasugian, Rita Marleta Dewi, Sarwo Handayani, Masri Sembiring Maha, Firda Fairuza, Meiriani Sari, Dita Setiati, Christina Safira Whinie Lestari

**Affiliations:** 1 Center for Biomedical Research, Research Organization for Health, National Research and Innovation Agency (BRIN), Bogor, West Java, Indonesia; 2 Department of Biology, Faculty of Medicine, Universitas Trisakti, West Jakarta, Indonesia; 3 USAKTI Center for Artificial Intelligence and Advanced Technology (CAPTIVATE), Jakarta, Indonesia; 4 Department of Paediatrics, Faculty of Medicine, Universitas Trisakti, West Jakarta, Jakarta, Indonesia; 5 Research Center for Preclinical and Clinical Medicine, Research Organization for Health, National Research and Innovation Agency (BRIN), Bogor, West Java, Indonesia; Universidad Nacional de la Plata, ARGENTINA

## Abstract

*Bordetella pertussis* infection is a highly contagious respiratory disease that can cause complications such as pneumonia and death. A total of 62,646 cases of pertussis worldwide were reported by WHO in 2022. This study aimed to obtain the pertussis seroprevalence and sociodemographic data in children aged 1–14 years and its association factors in the community based on Riskesdas 2013 and 2018. Bivariate and multivariate analysis was carried out on data from 12,753 children aged 1–14 years collected from Riskesdas 2013 and 2018 in Indonesia. Pertussis serology data was obtained based on the results of the ELISA examination which was categorized as seropositive if anti-pertussis toxin IgG ≥ 100 IU/mL or anti-pertussis IgG > 11 NTU. Pertussis seropositive indicated recent pertussis infection if no pertussis vaccine was received within the last twelve months. Pertussis seroprevalence was found at 9.8% and 33.4% in Riskesdas 2013 and 2018 respectively. While 10.1% of children aged 5–14 years were found pertussis seropositive by excluding the possible effect of vaccination in the last twelve months in Riskesdas 2013. The most important associated factor in seropositive pertussis at ages 1–4 years and 5–14 years was a history of pneumonia in the last month (OR = 2.709, 95%CI: 2.592–2.831 in Riskesdas 2013 and OR = 2.421, 95%CI: 2.299–2.550 in Riskesdas 2018). In the adjusted analysis for respondents’ characteristics, low maternal education was the predictive factor that most influenced pertussis seropositivity, especially in the 2013 Riskesdas (APOR = 2.983, 95%CI: 2.670–3.333). In conclusion, the results of this study showed that the seroprevalence of pertussis was high, especially in children aged 5–14 years, so that pertussis vaccine booster administration could be considered. Because the most influencing factor towards pertussis seropositive was low maternal education, the groups of children with low-educated mothers should be targets for strengthening complete vaccination coverage and disease control.

## Introduction

Pertussis is caused by *Bordetella pertussis*, also known as whooping cough, which is a highly contagious respiratory ailment [1]. Symptoms usually develop 7 to 10 days after infection. They include a mild fever, runny nose, and cough, which in most cases progresses to a hacking cough followed by whooping (hence the common name whooping cough). Pneumonia is a common complication, while seizures and brain disease seldom occur [2, 3]. The recurrence of newborn pertussis is of particular concern since infants are most vulnerable to severe morbidity and mortality, especially before they complete their first pertussis immunization series [4]. The World Health Organization (WHO) reported 62,646 cases of pertussis worldwide in 2022, China had the highest number of cases (38,295) [5]. A low number of pertussis cases were reported in Indonesia recently. However, there were more than 1,000 cases reported in Indonesia in 2017, 2014, and 2013 [6, 7]. Apart from that, pertussis cases in the field are considered to be higher than those reported based on the Indonesian Ministry of Health (Kemenkes RI) data and previous studies [7, 8].

Immunization is the most effective method of preventing pertussis. The incidence of severe pertussis in infants is reduced by the three-dose primary series diphtheria-tetanus-pertussis (DTP3) vaccinations [2]. In 2021, WHO reported about 81% (105 million infants) of the target population worldwide have gotten the advised three doses of the DTP-containing vaccination during infancy [9]. Indonesia has implemented the Expanded Program Immunization (EPI) basic immunization program for diphtheria, pertussis, and tetanus (DTwP) since 1976 with high immunization coverage for infants. Since 2018, the Indonesian Ministry of Health (Kemenkes RI) has changed the concept of complete basic immunization to complete routine immunization. Pertussis-containing vaccine is given in three doses to infants 2, 3, and 4 months, then 1 booster dose is added at 18 months, for continued immunization. The pertussis-containing vaccine in Indonesia is given in the form of whole cell pertussis vaccine (wP) and combined with diphtheria toxoid, tetanus toxoid, hepatitis B, and *Haemophilus influenzae b* [10, 11].

The evaluation of preventable infection diseases, vaccination achievements, and child immunity regularly, is very important for global vaccine policy information and improvement of program implementation, towards the targets set by the Global Vaccine Plan (GVAP) and the 2030 Immunization Agenda [12, 13]. The Indonesian Ministry of Health (Kemenkes RI) carried out basic health research (Riskesdas) in 2013 and 2018 which provided data on immunization coverage and serology for several diseases that can be prevented by immunization, including pertussis. The data on pertussis seropositive was available in children 1–14 years old which represented the national data of Indonesia with a large-scale survey sample in the community. The objective of this study was to estimate the seroprevalence of pertussis and sociodemographic data of children aged 1–14 years in the community and its association factors based on Riskesdas 2013 and 2018 data. The information is important as evidence-based in formulating an effective immunization strategy in the future.

## Materials and methods

### Study design and samples

This research is a further analysis of the data collected in the Riskesdas 2013 and 2018. Data were obtained from the Centre of Data and Information, of the Health Development Policy Agency, Ministry of Health, Indonesia [14]. The Riskesdas was carried out periodically every five years using a cross-sectional design, which represented the community on a national scale. The data provided consists of unique ID numbers for each research subject, which were not linked to individual identity data, ensuring the anonymity of the research subjects’ data. The outline of samples was planned based on calculations from the Indonesian Central Statistics Agency (BPS) by implies of multistage sampling through the stages as detailed in the national reports of the Riskesdas 2013 and 2018 [15–17]. Data from structured interviews and measurements on selected household and individual samples were then subjected to further analysis. The samples used for further analysis were children 1–14 years old who had undergone serological examination.

Blood samples in the Riskesdas 2013 and 2018 were taken from healthy individuals in the community, from all provinces in Indonesia, aged one year and above, and who met the inclusion criteria during the survey period. However, serological testing for anti-Pertussis IgG was only performed on children aged 1–14 years to assess immune response and the risk of pertussis infection, which would be linked to data on immunization history, history of acute respiratory infection, and pneumonia. In Riskesdas 2013, of the 7,229 blood serums of children aged 1–14 years examined for anti-PT antibodies, there were 6,542 (90.5%) who had completed the questionnaire data. In addition, the anthropometry measurements were complete and could be linked to the examination data ELISA for analysis. Meanwhile, at Riskesdas 2018, of the 7,032 tested for anti-Pertussis IgG antibodies, there were 6,211 (88.3%) who met the requirements for analysis. Out of the total samples, the toddler samples aged 1–4 years old were 568 (8.6%) children (in 2013) and 992 (15.97%) children (in 2018). The sample represented urban and rural areas nationwide [16, 17].

Pertussis serology data was obtained from the results of the Enzyme-Linked Immunosorbent Assay (ELISA) examination conducted at the research laboratory of the National Institute of Health Research and Development, Indonesian Ministry of Health. The serological examination of pertussis performed in the Riskesdas 2013 and 2018 used a semi-qualitative commercial ELISA with NovaLisa *B*. *pertussis* toxin (PT) IgG (Cat No; BPTG0610, NovaTec Immundiagnostica GmbH, Germany) and NovaTec *B*. pertussis IgG (Cat No; BOPG0030, NovaTec Immundiagnostica GmbH, Germany) reagents for the detection of anti-Pertussis IgG and was interpreted according to the manufacturer’s instructions. ELISA results were categorized as pertussis seropositive if anti-pertussis toxin IgG ≥ 100 IU/mL (in Riskesdas 2013) or anti-pertussis IgG > 11 NovaTec Unit (NTU) (in Riskesdas 2018). This parameter indicated a recent pertussis infection if no pertussis vaccine was received within the last twelve months.

### Operational definitions

Complete pertussis immunization for data analysis in the Riskesdas 2013 and 2018 namely three doses of pertussis vaccine given to infants 2, 3, and 4 months together with Hepatitis B vaccine in the form of the DTwP-HB combo (in 2011) which was replaced by DTwP-HB-Hib pentavalent (Biofarma Indonesia) in 2015 [18, 19]. Positive acute respiratory infection (ARI) and pneumonia based on the confession of a respondent or guardian, who in the last month, had a history of symptoms or had been diagnosed by a health worker. Economic status was measured by expenditure quintiles, namely the grouping of expenditures into five equal groups after being sorted from smallest to largest expenditure. Quintiles 1–2 were classified as poor, quintiles 3–4 as middle class, and quintile 5 as rich. The measurement of nutritional status in children 1–14 years was used based on the body mass index (BMI) for age. The BMI *Z*-score of undernutrition status is < -2.0; whereas the BMI *Z*-score of normal nutrition is +2.0 to -2.0; and overnutrition status means a combination of excess nutrition and obesity, with BMI *Z*-score > +2.0 [20–22]. Maternal education is categorized into 3 levels, namely low for those without school, medium for elementary to high school, and high for diploma-1 (D1) to college.

### Statistical analysis

At the time of analysis, authors used “weighting” which had been carried out by the Centre of Data and Information, of the Health Development Policy Agency, Ministry of Health, Indonesia. Descriptive analysis was carried out to identify the characteristics of the respondents and association factors of pertussis seropositivity, including age, sex, DTwP immunization status, history of acute respiratory infection, pneumonia, nutrition status, family economic level, and maternal education level. Sample grouping was performed on several research variables to answer the purpose of further analysis. Grouping for ages 1–4 and 5–14 years was carried out to minimize data bias due to the possible effect of vaccination on seropositivity at ages 1–4 years. The Prevalence Odds Ratio (POR) was assessed using the single logistic regression test. The multivariate test was performed to identify factors that associated with the seroprevalence of pertussis. All the unknown data was not included in the further analysis. The analysis was performed using StataCorp software version 16.4.

### Ethical consideration

This further analysis used secondary data, which was not linked to the research subjects, thus it did not require ethical clearance. Consent to utilize and analyze the dataset was obtained by submitting a research proposal to the Centre of Data and Information, the Health Development Policy Agency (Indonesia: Pusat Data dan Informasi, Badan Kebijakan Pembangunan Kesehatan), Ministry of Health, the Republic of Indonesia via email: datin.bkpk@kemkes.go.id.

## Results

The general characteristics of the respondents analyzed in the 2013 and 2018 Riskesdas are shown in Table 1. The respondents of the Riskesdas 2013 mostly lived in rural areas (51.3%), while in the Riskesdas 2018, respondents mostly lived in urban areas (66.5%). The proportion of respondents was higher in the boy group, aged 5–14 years, poor-middle economic status, normal nutrition, completed immunization status, and medium maternal education level both in Riskesdas 2013 and 2018. The proportion of toddlers (1–4 years old) with complete immunization status in the Riskesdas 2013 was 74.6%, meanwhile in the Riskesdas 2018 was 78.3%. Maternal education level was more commonly found in the middle category, followed by low and high education in each Riskesdas. Most respondents had no history of suffering from acute respiratory infection (ARI) or pneumonia in the last month (Riskesdas 2013 and 2018 data).

**Table 1:** Distribution of the respondents according to their characteristics in the Riskesdas 2013 and 2018.

Characteristic							
		Riskesdas 2013			Riskesdas 2018		
Demographic		n	N	Percentage (%)	n	N	Percentage (%)
Area							
	Urban	2,940	1,324,240	48.7	3,752	2,696,317	66.5
	Rural	4,170	1,394,848	51.3	3,451	1,357,397	33.5
Sex							
	Boy	3,664	1,401,863	51.6	3,815	2,094,626	51.7
	Girl	3,446	1,317,226	48.4	3,388	1,959,088	48.3
Age group (years old)							
	1–4	568	229,958	8.50	992	590,559	14.5
	5–14	6,542	2,489,131	91.50	6,211	3,463,155	85.5
Family economy status							
	Poor	2,980	1,003,564	36.9	3,012	1,586,223	45.8
	Middle class	3,061	1,296,653	47.7	2,264	1,278,372	36.9
	Rich	1,069	418,871	15.4	919	600,336	17.3
	Unknown	0			1,008		
Nutritional status							
	Undernutrition	640	214,351	9.19	543	295,108	7.3
	Good nutrition	5,138	1,984,818	85.08	6,016	3,391,459	84.3
	Overnutrition	346	133,776	5.73	583	335,289	8.3
	Unknown	986			61		
Complete DTP immunization status (1–4 years old)							
	Complete	375	151,225	74.6	658	386,418	78.3
	Uncomplete/Unknown	110	51,408	25.4	167	107,222	21.7
	Unknown	83			167		
Acute Respiratory Infection in last month							
	Yes	2,044	804,431	29.6	892	536,626	13.2
	No	5,065	1,914,253	70.4	6,311	3,517,088	86.8
	Unknown	1			0		
Pneumonia in last month							
	Yes	336	143,017	5.3	25	16,340	0.4
	No	6,754	2,567,503	94.7	7,047	3,964,716	99.6
	Unknown	20			131		
Maternal Education Level							
	Low	1,153	394,894	16.1	796	428,071	11.6
	Medium	4,916	1,966,464	80.2	5,270	3,046,483	82.52
	High	255	90,821	3.7	408	217,269	5.89
	Unknown	786			729		

n: unweighting samples, N: weighting samples

Since pertussis is a vaccine-preventable disease, we conducted an analysis of pertussis seropositivity in relation to the immunization status of respondents, which was only available for the 1–4 years age group. Table 2 showed the results of statistical analysis on pertussis seropositive respondents with complete DTwP immunization status. Pertussis seropositive was found in 9.8% respondents which 7.4% were at aged 1–4 years and 10.1% at aged 5–14 years in Riskesdas 2013 subjects. Meanwhile, in the Riskesdas 2018, seropositive pertussis was found in 33.4% of respondents, which 49.3% were aged 1–4 years and 30.7% were aged 5–14 years. In both Riskesdas 2013 and 2018, the complete DTwP immunization status based on age showed a similar proportion, with the highest being at one year of age. In Riskesdas 2013, respondents aged 1–4 years with a complete DTwP immunization history, the highest proportion of seropositive pertussis was in children aged one year (14.3%). Meanwhile, in Riskesdas 2018, the highest proportion of seropositive pertussis was at the age of four years (60.3%) (Table 2).

**Table 2:** The pertussis seropositive in respondents with complete immunization based on age*.

	Age (Years Old)	Riskesdas 2013				Riskesdas 2018			
		n	N	%	POR (95%CI)	n	N	%	POR (95%CI)
Pertussis Seropositive									
	1–4	568	229,958	7.40	ref	991	590,060	49.30	ref
	5–14	6,542	2,489,131	10.10	1.394 (1.372–1.416)	6,207	3,461,040	30.70	0.456 (0.453–0.459)
	Total	7,110	2,719,089	9.80	-	7,203	4,051,100	33.40	-
Complete DTwP immunization status									
	1 y.o	72	31,876	82.60	ref	184	107,109	85.80	ref
	2.y.o	111	45,955	80.50	0.865 (0.833–0.898)	189	127,119	81.90	0.748 (0.732–0.765)
	3 y.o	131	54,913	70.10	0.492 (0.475–0.509)	226	127,435	73.70	0.463 (0.453–0.473)
	4 y.o	171	69,889	70.70	0.508 (0.491–0.525)	226	131,977	73.10	0.448 (0.439–0.458)
Pertussis seropositive in respondent with complete immunization									
	1 y.o	53	26,339	14.30	1.899 (1.714–2.103)	158	91,427	49.10	1.191 (1.150–1.233)
	2.y.o	91	36,973	2.30	0.520 (0.460–0.588)	155	103,129	53.40	1.247 (1.212–1.284)
	3 y.o	100	38,474	9.30	0.592 (0.560–0.625)	182	93,936	48.10	1.547 (1.508–1.587)
	4 y.o	131	49,439	5.20	0.570 (0.535–0.606)	162	96,427	60.30	1.344 (1.312–1.378)

*Simple Logistic Regression Test, n: unweighting samples, N: weighting samples, POR: Prevalence Odds Ratio

Furthermore, for determining the relationship between pertussis seroprevalence and respondent characteristics in all data of Riskesdas 2013 and 2018, multivariate analysis was conducted. Results showed that low maternal education was the predictive factor that most influences pertussis seropositivity after other variables were controlled, especially in Riskesdas 2013 (APOR = 2.983, 95% CI: 2.670–3.333) (Table 3).

**Table 3:** The association between pertussis seroprevalence and respondent characteristics in Riskesdas 2013 and 2018^#^.

Characteristics		n	% Positive	% Negative	POR (95%CI)	p	APOR (95%CI)	p
								
**Riskesdas 2013**								
Area								
	Urban	2,940	9.7	90.3	1.037 (1.029–1.046)	0.713	1.142 (1.095–1.190)	0.000
	Rural	4,170	10	90	ref			
Sex								
	Girl	3,446	9.7	90.3	1.024 (1.016–1.032)	0.000	0.588 (0.567–0.610)	0.000
	Boy	3,664	9.9	90.1	ref			
Age Group (years old)								
	1–4	568	7.4	92.6	1.394 (1.372–1.416)	0.000		
	5–14	6,542	10.1	89.9	ref			
Family Economy Status								
	Poor	2,980	11.5	88.5	1.042 (1.030–1.054)	0.000	0.96 (0.907–1.016)	0.161
	Middle Class	3,061	8.1	91.9	0.707 (0.696–0.715)		0.773 90.688–0.760)	0.000
	Rich	919	33.7	66.1	ref			
Nutritional Status								
	Undernutrition	640	9.4	90.6	1.084 (1.067–1.1)	0.000	0.301 (0.254–0.357)	0.000
	Normal Nutrition	5,138	10.1	89.9	0.928 (0.906–0.951)	0.000	1.817 (1.681–1.963)	0.000
	Overnutrition	346	8.8	91.2	ref			
Complete DTP Immunization Status (1–4 years old)								
	Incomplete/Unknown	110	9.8	90.2	1.422 (1.373–1.472)	0.000	1.539 (1.482–1.599)	0.000
	Complete	375	7.1	92.9	ref			
History of Acute Respiratory Infection in the Last Month								
	Yes	2,044	10	90	1.022 (1.013–1.031)	0.000	1.109 (1.068–1.152)	0.000
	No	5,065	9.8	90.2	ref			
History of Pneumonia in the Last Month								
	Yes	336	13.1	86.9	1.410 (1.387–1.432)	0.000	1.979 (1.887–2.086)	0.000
	No	6,754	9.6	90.4	ref			
Maternal Education Level								
	Low	1,153	10.92	89.08	1.226 (1.196–1.257)	0.007	2.983 (2.670–3.333)	0.000
	Medium	4,916	9.8	90.2	1.086 (1.061–1.112)	0.000	1.383 (1.215–1.531)	0.000
	High	255	9.09	90.91	ref			
**Riskesdas 2018**								
Area								
	Urban	3,752	33.4	66.6	0.999 (0.995–1.003)	0.000	1.226 (1.209–1.244)	0.000
	Rural	3,446	33.4	66.6	ref			
Sex								
	Female	3,812	33.1	66.9	1.029 (1.025–1.034)	0.000	1.096 (1.083–1.111)	0.000
	Male	3,386	33.8	66.2	ref			
Age Group (years old)								
	1–4	991	49.3	50.7	0.456 (0.454–0.459)	0.000		
	5–14	6,207	30.7	69.3	ref			
Family Economy Status								
	Poor	3,010	31.5	68.5	0.912 (0.906–0.918)	0.000	0.697 (0.684–0.684)	0.000
	Middle Class	2,261	36.5	63.5	1.141 (1.134–1.149)		1.135 (1.114–1.157)	0.000
	Rich	919	33.7	66.1	ref			
Nutritional Status							
	Undernutrition	543	37.4	62.6	0.597 (0.593–0.601)	0.000	1.042 (1.009–1.077)	0.000
	Good Nutrition	6,012	32.9	67.1	0.489 (0.488–0.490)	0.000	1.463 (1.429–1.499)	0.000
	Overnutrition	582	34.8	65.2	ref			
Complete DTP Immunization Status (1–4 years old)								
	Incomplete/Unknown	167	45.9	54.1	0.758 (0.748–0.768)	0.000	0.855 (0.842–0.868)	0.000
	Complete	657	52.8	47.2	ref			
Acute Respiratory Infection in the Last Month								
	Yes	891	31.8	68.2	0.918 (0.912–0.924)	0.000	0.732 (0.719–0.746)	0.000
	No	6,307	33.7	66.3	ref			
Pneumonia in the Last Month								
	Yes	25	36.9	63.1	1.155 (1.119–1.192)	0.000	0.613 (0.582–0.646)	0.000
	NO	7,042	33.6	66.4	ref			
Maternal Education Level								
	Low	795	33.1	66.9	1.015 (1.004–1.026)	0.007	1.181 (1.751–1.879)	0.000
	Medium	5,268	32.3	67.7	0.927 (0.92–0.937)	0.000	1.343 (1.309–1.378)	0.000
	High	407	33.7	66.3	ref			

^#^Multiple Regression Logistic Test, n: unweighting samples, POR: Prevalence Odds Ratio, APOR: Adjusted Prevalence Odds Ratio.

Table 4 shows the multivariate analysis that was carried out in the Riskesdas 2013 and 2018 data to determine the association factors of pertussis seroprevalence with respondent characteristics. Data were divided into two groups based on age 1–4 and 5–14 years to distinguish complete DTwP immunization history data which was only available in children aged 1–4 years. The results of this study showed that several factors such as history of pneumonia in the last month (POR = 2.709, 95%CI: 2.592–2.831), history of acute respiratory infection in the last month (POR = 2.043, 95%CI: 1.980–2.108) and nutrition status (POR = 2.186, 95%CI: 1.978–2.416) were the factors that played the most role in differentiating the incidence of seropositive pertussis in children aged 1–4 years and 5–14 years compared to other variables in the Riskesdas 2013. Meanwhile, in Riskesdas 2018, the history factor pneumonia in the last month (POR = 2.421, 95%CI: 2.299–2.550) played a greater role compared to other variable factors, when observed from the prevalence odds ratio value. In addition, the history of pneumonia and history of acute respiratory infection in children with seropositive pertussis was higher in children aged 1–4 years compared to those aged 5–14 years, both in Riskesdas 2013 and 2018. Although the results of the statistical analysis also indicated the role of nutrition in the immune response to vaccination, which was indicated by the high proportion of pertussis seropositive in children aged 1–14 years with good nutrition compared to malnutrition. However, after adjusting for other variables, the history of pneumonia played a more important role (Table 4).

**Table 4:** The association between respondent characteristics and pertussis seropositive based on age groups 1–4 years and 5–14 years in Riskesdas 2013 and 2018.

		Riskesdas 2013							Riskesdas 2018						
		n	1–4	5–14	P	POR (95%CI)	APOR (95%CI)	P	n	1–4	5–14	P	POR (95% CI)	APOR (95%CI)	p
Area															
	Urban	268	48.4	47.8	0.139	1.024 (0.992–1.056)	0.770 (0.742–0.799)	0.000	1209	72.8	64.9	0.000	1.448 (1.435–1.461)	1.392 (1.378–1.407)	0.000
	Rural	420	51.6	52.2					1144	27.2	35.1				
Sex															
	Female	346	40.7	48.4	0.000	0.732 (0.710–0.756)	0.652 (0.630–0.676)	0.000	1124	47.1	52.3	0.000	0.812 (0.806–0.819)	0.786 (0.779–0.793)	0.000
	Male	342	59.3	51.6					1229	52.9	47.7				
History Pneumonia in the Last Month															
	Yes	37	15.7	6.4	0.000	2.709 (2.592–2.831)	1.772 (1.683–1.865)	0.000	5	0.8	0.3	0.000	2.421 (2.299–2.550)	1.878 (1.782–1.978)	0.000
	No	647	84.3	93.6					2312	99.2	99.7				
History Acute Respiratory Infection in the Last Month															
	Yes	188	45.4	29.0	0.000	2.043 (1.980–2.108)	1.994 (1.925–2.066)	0.000	274	14.1	12.2	0.000	1.190 (1.176–1.204)	1.233 (1.217–1.250)	0.000
	No	500	54.6	71.0					2079	85.9	87.8				
Family Economic Status															
	Poor	327	38.8	43.5	0.000	1,233 (1,181–1,288)	0.451 (0.428–0.476)	0.000	949	39.4	43.7	0.000	1.321 (1.305–1.337)	0.904 (0.891–0.917)	0.000
	Medium	258	42.2	39.2	0.385	1,019 (0.976–1,064)	0.622 (0.593–0.653)	0.000	777	40.9	39.7	0.000	1.158 (1.143–1.172)	0.920 (0.908–0.933)	0.000
	Rich	103	19.0	17.3		ref			303	19.7	16.6				
Nutrition Status															
	Undernutrition	58	4.6	9.0	0.000	2,186 (1,978–2,416)	0.398 (0.356–0.445)	0.000	191	7.2	8.5	0.000	1.043 (1.022–1.066)	0.944 (0.922–0.966)	0.000
	Normal nutition	506	89.9	86.0	0.079	1,065 (0.993–1,142)	1.174 (1.087–1.269)	0.000	1939	85.0	82.6	0.000	0.851 (0.838–0.865)	1.124 (1.105–1.143)	0.000
	Overnutrition	36	5.6	5.0		ref			194	7.8	8.9				
Maternal Education Level															
	Low	118	24.0	17.2	0.000	1.226 (1.196–1.257)	1.185 (1.062–1.323)	0.003	263	7.7	13.5	0.000	0.418 (0.409–0.427)	0.538 (0.525–0.551)	0.000
	Medium	481	73.2	79.4	0.000	1.087 (1.062–1.112)	0.902 (0.814–1.001)	0.052	1703	84.6	80.9	0.000	0.767 (0.753–0.778)	0.829 (0.813–0.845)	0.000
	High	20	2.8	3.4		ref			137	7.7	5.6				

n: unweighting samples, POR: Prevalence Odds Ratio, APOR: Adjusted Prevalence Odds Ratio.

## Discussion

Riskesdas is a periodic national health survey every five years that collects abundant variable data, which aims to provide an overview of the health status of the Indonesian population during the survey period. So, there were limited data that could be analyzed for this study, not all the required variables were available, including the history of pertussis vaccination for children aged 5–14 years. In addition, in 2017–2018, outbreak response immunization (ORI) was carried out with the DTwP vaccine in children aged 1–5 years due to the diphtheria outbreak in Indonesia. Unfortunately, there was no data related to information about ORI recipients in the Riskesdas 2018. This might lead to bias in determining whether the increase in pertussis IgG was a result of vaccination or natural infection, particularly in Riskesdas 2018 data. In addition, although the pertussis serological examination method used in the Riskesdas 2013 and 2018 was similar (ELISA). However, the kits were different, including the cut-off values and seropositivity units.

The age group was divided into two (1–4 years and 5–14 years) because data on immunization history was only available for children aged 1–4 years. Specifically, the Riskesdas 2013 data could provide an overview of pertussis infection in children aged 5–14 years, because there was no history of pertussis vaccination in the last twelve months. This is related to the routine immunization program for pertussis in Indonesia involved administering three doses of the DTP vaccine, in the form of a whole-cell pertussis; at two, three, and four months of age. However, since 2018, a booster dose of the DTP vaccine at 18 months of age was included in the immunization program [7, 10, 11]. In the Riskesdas 2013 and 2018 data, the routine immunization program of the Indonesian government for pertussis still consisted of three doses of the DTP vaccine. However, from late 2017 to 2018, there was additional DTwP vaccination for children aged 1–5 years in response to the diphtheria outbreak, so it was likely to influence the seropositive pertussis data in the Riskesdas 2018. During the year 2017, Indonesia experienced a diphtheria outbreak in 170 districts/cities and 30 provinces, with a total of 954 cases and 44 deaths. Consequently, an outbreak response immunization (ORI) program was implemented to break the chain of transmission through vaccination. The vaccination containing pertussis was provided in the ORI program in the form of pentabio vaccine (DTP-HB-Hib vaccine), targeting children aged 1–5 years. The ORI program started in December 2017 in 12 districts/cities and continued gradually in 73 districts/cities across 11 other provinces in 2018 [23]. The addition of pertussis-containing vaccinations since 2017, it might affect the results of anti-pertussis Toxin IgG in the Riskesdas 2018 [24, 25].

The studies conducted by Alexandre Pereira *et al*. and Nasamon Wanlapakorn *et al*. in Brazil and Thailand also showed that children with a history of complete DTwP immunization still exhibited high levels of serum antibodies against pertussis at least two years after vaccination. This is because the vaccine induces an effective immunologic memory [26, 27]. Therefore, the increase in IgG at the age of 1–4 years could be caused by both vaccination and natural infection. Meanwhile, an increase in IgG > 100 IU at the age of 5–14 years could almost certainly be caused by infection, particularly in the Riskesdas 2013.

The high levels of Ig-PT antibodies (≥100 IU/mL) in age groups not targeted for vaccination could indicate a higher likelihood of natural pertussis infection [28]. In 2013, the prevalence of pertussis in children aged 5–14 years in Indonesia was estimated at 10.1% based on seropositive pertussis data (Table 2). This number was much higher than the case numbers reported by WHO (27 cases in 2019 and 36 cases in 2020) and the Ministry of Health, Indonesia (386 cases in 2019) [6, 7]. This was due to the general similarity of clinical manifestation of pertussis with ARI and some cases were mild or asymptomatic [29, 30] so they were not reported as pertussis. Therefore, a booster pertussis vaccine should be considered to prevent pertussis infection, since currently, the national pertussis vaccination program is up to 18 months of age. This is also in accordance with WHO recommendations, with a four doses pertussis vaccine administration schedule, it can provide protection for at least six years for countries that use the wP vaccine [31]. Thus, further analysis of the 2023 Riskesdas data is warranted to assess the impact of the pertussis vaccination booster at 18 months of age on the prevalence of pertussis infection. A study conducted in Thailand on 900 serum samples showed that after the age of 11 years, the highest proportion of anti-PT IgG >100 IU/ml was observed [27]. Furthermore, a study in China involving 2144 subjects aged three days to 76 years showed that the highest percentage of recent pertussis infection (anti-PT IgG >100 IU/ml) was observed in children aged nine years (5.1%) (95% CI 0.0–12.1) [32]. The high number of unreported cases of pertussis needs attention because pertussis transmission can occur without any contact with sufferers who are ’diagnosed’ with pertussis. The high rate of subclinical infections among household contacts of pertussis sufferers, might be an important factor in disease transmission [30].

In Table 2, for children aged 1–4 years with a complete history of DTwP immunization, the highest proportion of pertussis seropositive was aged one year in the Riskesdas 2013, because there was a history of getting pertussis vaccination in babies according to the government’s routine vaccination program. Meanwhile, in the Riskesdas 2018, the highest proportion was children aged 4 years, possibly due to additional vaccination given in the form of the DTwP vaccine as a response to the diphtheria outbreak, although this could not be confirmed due to limited data available.

Apart from that, the use of commercial kits with NTU units in the Riskesdas 2018 raised concerns about differences in results from commercial kits with IU units used in the Riskesdas 2013. Even though this study used the same categories (seropositive and seronegative), in reality, no references were found, which compared the results of both types of commercial kits on the same samples. This was supported by evidence that in general, the prevalence of pertussis seropositive was much greater in Riskesdas 2018 compared to Riskesdas 2013, even at ages 5–14 years, where the influence of ORI could be minimized (Table 2). No strong data was obtained to confirm whether the high proportion of pertussis seropositive in the Riskesdas 2018 was caused by high levels of infection, the effect of vaccination (ORI), or the commercial kits used.

Based on multivariate analysis, the predictive factor that most influences the pertussis seropositivity of characteristics of respondents was low maternal education (Table 3). Although maternal education is not directly related to the incidence of pertussis, maternal educational status is directly related to the completeness of vaccination, which can have an impact on vaccine-preventable disease infections, including pertussis. We added an analysis of the relationship between completeness of vaccination and maternal education level, showing that completeness of vaccination was 5.48 times higher (95% CI 5.03–5.07) in 2013 Riskesdas and 1.38 times higher (95% CI 1.33–1.43) in 2018 Riskesdas in higher maternal education level groups compared to low maternal education group (data not shown). An analysis survey conducted in Eritrea showed that children of mothers with no education had lower full vaccination coverage than those with middle or above education (OR = 2.34, 95% CI 1.30–4.21) [33]. Another study on infectious diseases in Bangladesh in the form of a nationwide cross-sectional survey showed results similar to our study, that children with mothers with no education, low and moderate education showed higher odds of acute respiratory infection than mothers with higher education [34]. Therefore, groups of mothers with low education or no education should become targets for strengthening vaccination programs and controlling infectious diseases.

In the 2013 Riskesdas data, a history of acute respiratory infection and pneumonia in the past month was the most significant factor for pertussis infection after controlling for other variables, while in the 2018 Riskesdas data, only a history of pneumonia was significant (Table 4). These findings suggested the possibility that pertussis infection increases the risk of complications leading to pneumonia. Pertussis (whooping cough) is a severe acute respiratory infection caused by the bacterial pathogen *B*. *pertussis*, with persistent coughing in children, adolescents and adults with severe respiratory illness [35]. Clinical manifestations of *B*. *pertussis* infection range from a relatively moderate cough condition to a serious and potentially deadly condition with pneumonia, convulsions, encephalopathy, and respiratory failure [36]. Co-infection cases with multiple pathogen species are becoming common and revealed as important issue for diseases development and treatment. In the upper respiratory tract, where exposure to microbial species is common, interactions between pathogens, often host-mediated, are of particular relevance. The co-infection between bacteria and influenza causing infection in the respiratory tract is an example of the well-studied interspecies interaction in co-infection cases. An example of an interaction between influenza virus, is their interaction with bacterial pathogens, including *Streptococcus pneumoniae*, *Streptococcus pyogenes*, *Staphylococcus aureus*, *Haemophilus influenza*, and *B*. *pertussis* [37]. These co-infections still play an important role today, as evidenced by the increased mortality associated with bacterial infections during recent influenza epidemics and pandemics.

There are several of studies aimed at explaining the clinical importance of the interaction occurred between influenza virus with bacterial pathogens in infected hosts, especially with *B*. *pertussis* [38, 39]. Seasonal influenza and pertussis infections are recognized as the leading causes of neonatal and infant morbidity and mortality worldwide [40, 41]. A study involving seven African and Asian countries in low- and middle-income regions (LMIC) on children aged 1–59 months, who were hospitalized with severe or very severe pneumonia, revealed the detection of *B*. *pertussis* in 53 out of 4200 (1.3%) cases. Pertussis-infected pneumonia cases in children aged 1–5 months exhibited a case fatality ratio of 12.5% (95% CI: 4.2%-26.8%; 5/40) [42]. The findings in our study, as well as other studies conducted in 7 countries across Asia and Africa, indicated the need for attention to pertussis, particularly in children, due to its potential for severe outcomes, including fatal cases.

## Conclusions

Further analysis from Riskesdas 2013 and 2018 data performed in this study revealed that the seroprevalence of pertussis in children aged 1–14 years was high, particularly those children aged 5–14 years. So, giving a pertussis vaccine booster needs to be considered. This was because at the time the Riskesdas 2013 and 2018 were implemented, the pertussis immunization program in Indonesia was still implementing three basic vaccination doses, and a new booster was implemented in 2018. Considering that maternal education is the characteristic factor most related to pertussis seroprevalence, the group of children and mothers with low education can be the main targets for strengthening complete vaccination coverage and efforts to control diseases, one of which is pertussis which can cause complications from pneumonia.
